# Cetacean Stranding Response Program and Spatial–Temporal Analysis in Taiwan, 1994–2018

**DOI:** 10.3390/ani14121823

**Published:** 2024-06-19

**Authors:** Lien-Siang Chou, Chiou-Ju Yao, Ming-Chih Wang, Wei-Lien Chi, Yun Ho, Wei-Cheng Yang

**Affiliations:** 1 Institute of Ecological and Evolutionary Biology, National Taiwan University, Taipei 106216, Taiwan; chouls@ntu.edu.tw (L.-S.C.); jj787834@gmail.com (Y.H.); 2 Department of Biology, National Museum of Natural Science, Taichung 404023, Taiwan; 3 Department of Life Science, National Chung Hsing University, Taichung 402202, Taiwan; 4 Taiwan Cetacean Society, New Taipei 248020, Taiwan; carl.7111@gmail.com (M.-C.W.); chiita.tw@gmail.com (W.-L.C.); 5 Department of Post-Baccalaureate Veterinary Medicine, College of Medical and Health Science, Asia University, Taichung 413305, Taiwan; 6 School of Veterinary Medicine, National Taiwan University, Taipei 106216, Taiwan

**Keywords:** stranding, cetaceans, Taiwan, western Pacific, spatial–temporal analysis

## Abstract

**Simple Summary:**

This study reviews the development of the cetacean stranding response program in Taiwan from 1994 to 2018, and examines 1320 stranding events involving 1698 animals across at least 27 species. The stranding rates have increased significantly over the years, with a notable rise in annual events from 16 to over 90. Seasonal variation demonstrates higher stranding rates during the northeastern monsoon season. The study highlights the spatial distribution of strandings, with the highest frequency in northern Taiwan and mass strandings predominantly in the southwest. Additionally, live strandings accounted for 29.5% of events and 38.9% of animals. The rescue and rehabilitation of some individuals have been conducted, and 15 individuals have been released since 2000. The findings underscore the diversity and stranding patterns of cetaceans around Taiwanese waters, and provide valuable information for developing conservation strategies in the western Pacific.

**Abstract:**

A national cetacean stranding response program in Taiwan has evolved significantly in the past three decades. Initially co-ordinated by National Taiwan University from 1994, the program transitioned to the Taiwan Cetacean Society in 1999, and local governments took on a more prominent role after 2009. A comprehensive stranding database (1994–2018) has been maintained, which documented 1320 stranding events involving 1698 animals from at least 27 species. The most commonly stranded species include finless porpoises, bottlenose dolphins, *Kogia* spp., and Risso’s dolphins. The stranding rates varied annually and seasonally, with increases noted from an average of 16 events per year for the first 3 years to 44–58 events per year between 1997 and 2015, and a sharp rise to over 90 events per year for the period of the last three years. Seasonal variations were also significant, with higher stranding rates during the northeastern monsoon (NEM, October to next April) than that during southwestern monsoon (SWM, May to September). From the aspect of distribution, more frequent and even strandings occurred along the coast of northern Taiwan, while mass strandings were concentrated in the southwestern counties during NEM. Among all strandings, 390 events (29.5%) and 660 animals (38.9%) were live ones. Under great effort in rescuing and rehabilitating 52 cases, 15 cetacean individuals have been released since 2000. Additionally, there have been 56 mass strandings involving at least 11 species since 1994, predominated by pygmy killer whales, particularly during the NEM season along the southwest coast. This study not only contributes to our understanding of the stranding patterns and diversity of the cetaceans in Taiwan, but also provides valuable insights for future conservation strategies on cetaceans in the western Pacific.

## 1. Introduction

Human life is intrinsically connected to ocean ecosystems, yet the comprehensive monitoring of these environments is challenging due to limited accessibility, funding, and manpower. The vastness and depth of the oceans make it difficult to collect consistent and comprehensive data, which are essential for understanding and protecting these critical environments [1]. Additionally, the financial and human resources required to conduct extensive oceanographic research often exceed available budgets, limiting the scope and frequency of scientific investigations [2]. Cetaceans and other marine mammals, occupying the apex of marine food webs, are excellent indicators of ocean health. As top predators, these animals accumulate contaminants and pathogens from their prey, making them valuable for monitoring changes in the marine environment. Their health and occurring pattern can reflect the condition of the ecosystem, providing early warnings of ecological disruptions [3,4]. Moreover, marine mammals have long lifespans and wide-ranging habitats, allowing scientists to gather data over extended periods and across vast geographic areas [5]. Marine mammal strandings offer valuable opportunities to identify and investigate issues within marine ecosystems. A stranding event occurs when marine mammals are found ashore, either deceased or alive but unable to return to the water independently [6]. These events can provide critical insights into the causes of mortality and morbidity in marine mammal populations, such as disease outbreaks, toxic algal blooms, or human-induced injuries [6]. Analyzing stranded animals can also reveal information about prey availability, oceanographic conditions, and pollution levels.

Maintaining a detailed stranding database is crucial for understanding species occurrence, distribution, and potential abundance [7]. Such databases compile records of stranding incidents, including species identification, location, date, and cause of death, if known. This information is invaluable for tracking population trends, identifying conservation priorities, and formulating management strategies. Furthermore, strandings can provide insights into both oceanic and human health, given that marine mammals are considered sentinel species. They often bioaccumulate pollutants and pathogens, which can serve as indicators of environmental health risks that may also affect human populations [8]. However, responding to strandings and collecting data typically require significant time, effort, and financial resources. Taiwan has been fortunate to have established a long-term cetacean stranding response program since 1994, successfully shifting the perception of cetaceans from fishing targets to conservation priorities.

**Figure 1:**
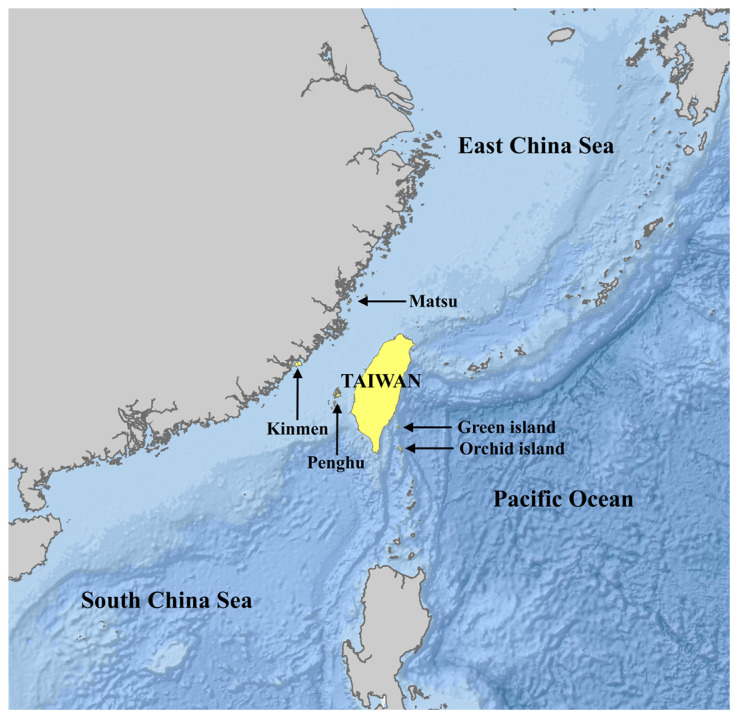
Geographical location of the study sites.

Taiwan’s geographical features, including its strategic location at the convergence of the Kuroshio Current and the South China Sea Current, contribute to its rich and diverse marine ecosystems. The island has bathymetric characteristics, such as its steep continental shelf off the east coast with deep oceanic trenches, and the shallow waters of the Taiwan Strait to the west coast (Figure 1), with five major isles including Penghu (P), Kinmen (K), Mazhu (M), Green (G), and Lanyu (L). These diverse habitats can support a wide array of cetacean species, from small dolphins to large baleen whales. Chou [9] reported that there were at least 29 species confirmed in Taiwanese waters. According to past cetacean sighting and stranding records, over 30 species of cetaceans have been recorded in Taiwan.

### 1.1. History of Cetacean Conservation and Stranding Response Program

Until 1990, cetaceans in Taiwan were merely considered part of the fishing harvest, and stranded cetaceans were either overlooked or used as a source of protein. That year, the Earth Trust, a US-based conservation NGO, screened a film in Hawaii depicting the dolphin slaughter off the Penghu Islands, which sparked significant backlash in the West. This led to widespread media coverage in Taiwan and subsequent discussions about the status of cetaceans, culminating in their inclusion under the Wildlife Conservation Act of 1989 as protected species. This marked a pivotal moment for the status of cetaceans and spurred both research and tourism focused on these marine mammals in Taiwan.

### 1.2. The Evolution of the Cetacean Stranding Program in Taiwan Is Outlined in Four Distinct Stages

1. Pioneer Stage (1990–1993)

Initiated by Richard Chen and his team at Ocean World, Taipei, this stage marked the beginning of active engagement with cetacean strandings, collecting news, and becoming involved in rescue operations. At this time, public knowledge about cetaceans was minimal.

2. Sprouting Stage (1994–1999)

LSC’s laboratory at National Taiwan University began systematically collecting data on stranded cetacean carcasses in 1994, collaborating with various agencies on biological studies of cetaceans. The increased media coverage of strandings raised public awareness and conservation efforts. LSC hosted over 14 symposia and numerous public education initiatives. In November 1996, the nationwide Taiwan Cetacean Stranding Network (TCSN) was established to enhance response and rescue efforts, initially co-ordinated by LSC at National Taiwan University, and later transitioning to the Taiwan Cetacean Society (TCS) in 1999.

3. NGO Leading Stage (2000–2008)

Supported by funding from the Forest Bureau, Council of Agriculture, this stage saw the expansion of the stranding program across Taiwan, including remote islands. The Taiwan Cetacean Society held annual meetings with government officials and collaborated with private rescue organizations and aquaria for short-term cetacean rehabilitation, although a long-term facility was yet to be developed. A comprehensive databank of stranding reports was created by MCW atop the initial decade of data collected by LSC. Since then, all local governments were requested to provide local stranding information, including those of the five major isles.

4. Governmental Leading Stage (2008–2018)

In 2008, the Forest Bureau restructured the roles among various agencies, enhancing the responsibilities of local governments. By late 2018, marine conservation efforts were centralized under the newly established Ocean Conservation Administration (OCA) within the Council of Ocean Affair, merging the existing network into the Marine Animal Rescue Network (MARN), led by OCA.

This chronological framework highlights the significant milestones and transitions in Taiwan’s approach to cetacean stranding and conservation efforts over nearly three decades. By analyzing the accumulated data on cetacean strandings in Taiwan, we seek to enhance our understanding of species distribution, to identify temporal and spatial patterns that may inform future conservation strategies and policy-making efforts, and to contribute to the global body of knowledge on marine mammal conservation.

## 2. Materials and Methods

The data for this study were sourced from the official TCSN database (https://www.whale.org.tw/portal_d828.php?owner_num=d828_565804&button_num=d828, non-public database, accessed on 1 March 2023) covering the years 1994 to 2018. The Excel spreadsheet database was meticulously reviewed on a case-by-case basis to correct any duplications or inconsistencies. This was supplemented with personal communications from the stranding team at the same institution and relevant published scientific studies. The study focused on species within the cetacean order, specifically the odontoceti and mysticeti suborders, which were identified in the database and used as the units of analysis. The selection criteria for each stranding case were based on the definition [10] where a “single stranding” is defined as either an individual animal or a mother/calf pair, and a “mass stranding” is defined as the simultaneous stranding of two or more cetaceans, excluding mother/calf pairs which are considered a single stranding.

Paired-sample *t*-test was performed to examine the difference between stranding trends during two monsoon seasons, including northeastern monsoon (NEM) from October to next April, and southwestern monsoon (SWM) from May to September [11]. The test was applied to both number of stranding events and number of stranded individuals. Statistical analysis was conducted with the software R version 4.3.2 [12]. As for spatial analysis, the distribution of total strandings over the 25-year dataset was visualized with ArcMap 10.7 with TWD97 as co-ordinate system (Esri Inc., Redlands, CA, USA). Kernel estimation was calculated using the tool Kernel Density (Spatial Analyst) with 15 km of search radius and 1 km as output grid size. Kernel Density tool calculated density of point data while considering neighboring points within a given distance [13]. This geoprocessing tool returned a raster with the expected number of stranding per square kilometer.

## 3. Results

From 1994 to 2018, a total of 1320 stranding events involving 1698 animals were reported (Table 1). At least 27 cetacean species have been recorded as stranded in Taiwan (Table 1), with Delphinidae being the most frequently encountered family, comprising 54.7% of the incidents. The species most commonly found stranded include finless porpoises (*Neophocaena* spp.), bottlenose dolphins (*Tursiops* spp.), *Kogia* spp., Risso’s dolphins (*Grampus griseus*), Fraser’s dolphins (*Lagenodelphis hosei*), and pantropical spotted dolphins (*Stenella attenuata*). Notably, the pygmy killer whale (*Feresa attenuata*) has the highest number of stranded individuals, despite ranking only seventh in event counts (Table 1).

**Figure 2:**
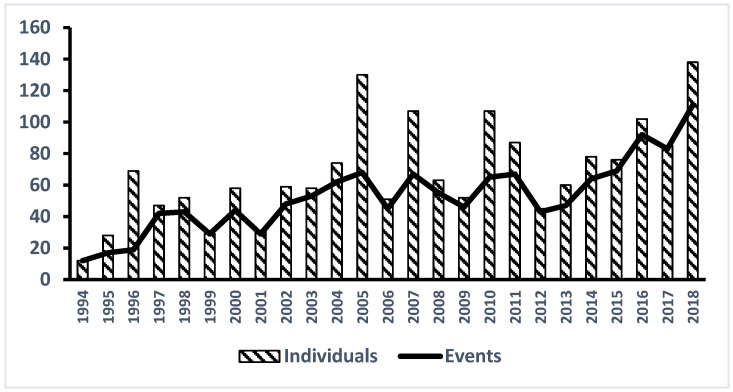
Annual variation of cetacean stranding events and individuals in Taiwan from 1994 to 2018, showing a total of 1320 events and 1698 individuals stranded.

**Figure 3:**
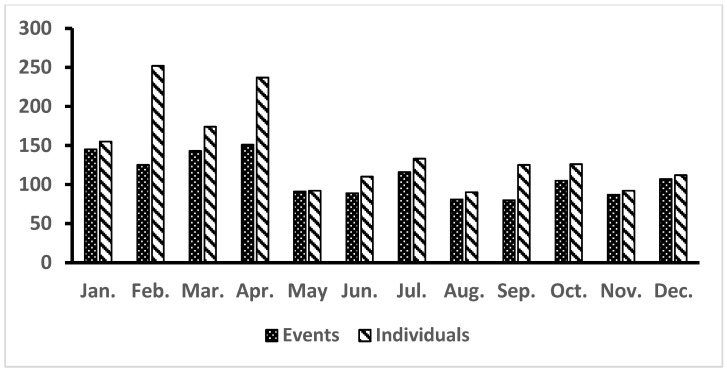
Monthly variation of cetacean stranding events and individuals in Taiwan from 1994 to 2018, illustrating a total of 1320 events and 1698 individuals stranded.

The average annual stranding frequency during the whole 25-year period was 52.8 events and 67.9 animals. Noticeably, the annual stranding rate exhibited a significant increasing trend in three distinct phases: initially, it rose from 16 events per year (1994–1996) to 41 events per year (1997–2003), then to 58 events per year (2004–2015), and, finally, surpassed 90 events per year in the last three years (Figure 2). Monthly fluctuations revealed a higher frequency of strandings from January to April, with accumulated events ranging from 125 to 151 (5–6 events per month), or accumulated individual animals ranging from 155 to 252 (6–10 animals per month) (Figure 3). The frequency of strandings reached the top in April and generally decreases during the summer months, reaching the lowest point in August, and then rises again in the autumn months. It demonstrated that the stranding frequency was higher during the NEM season (October to April) compared to the SWM season (May to September). This difference was statistically significant for both the number of stranding events and the number of animals involved (paired *t*-test, *p* < 0.05) (Figure 3). The discrepancy between the number of events and the number of animals suggests that mass strandings were more frequent during February to April and in September.

**Figure 4:**
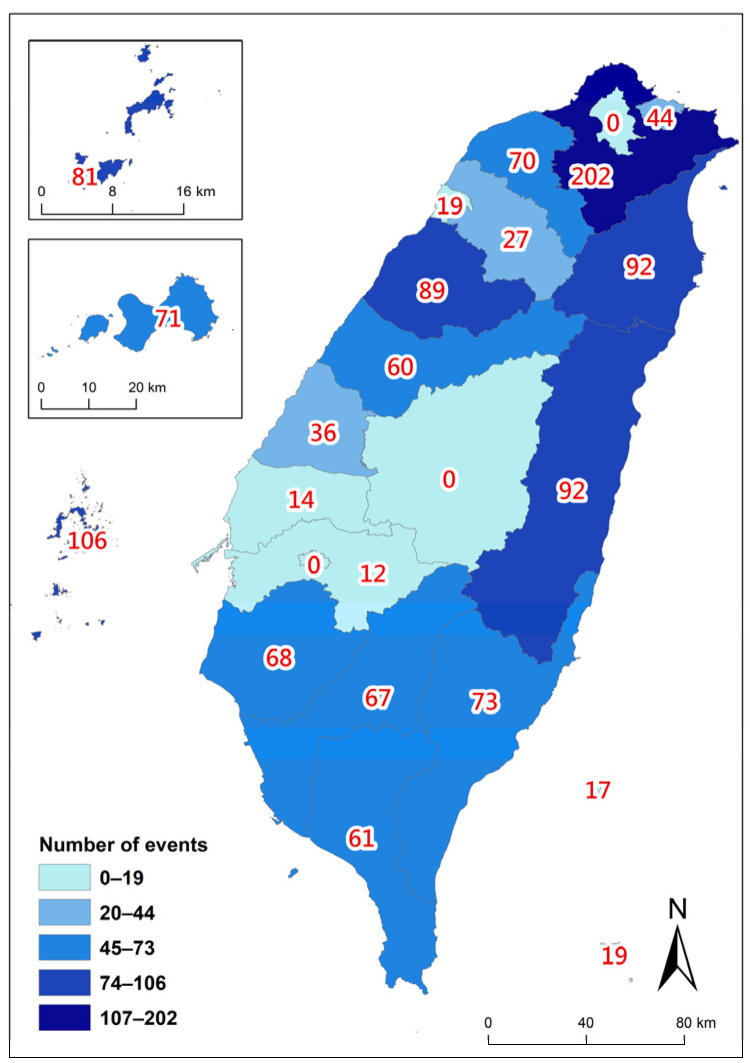
Spatial distribution of accumulated cetacean stranding events at various counties of Taiwan from 1994 to 2018.

**Figure 5:**
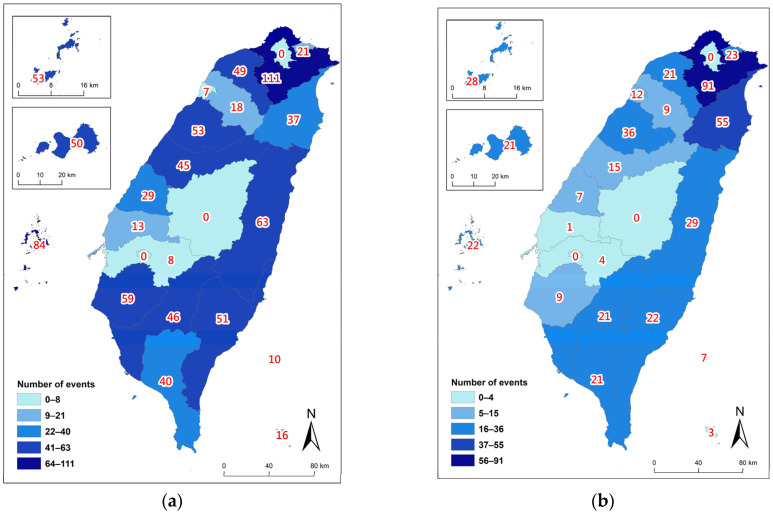
Spatial distribution of cetacean stranding events during (**a**) the Northeast Monsoon (NEM) season, and (**b**) the Southwest Monsoon (SWM) season at various counties of Taiwan from 1994 to 2018.

The spatial variation revealed a significant non-random distribution. Northeastern Taiwan recorded the highest number of stranding events, followed by counties in southern Taiwan and the outlying isles in the Taiwan Strait, respectively (Figure 4). Consistent with the previously mentioned higher stranding rates during the NEM compared to the SWM, all studied regions except for Northeastern Taiwan experienced a 2–6-fold increase in events during the NEM season (Figure 5).

**Figure 6:**
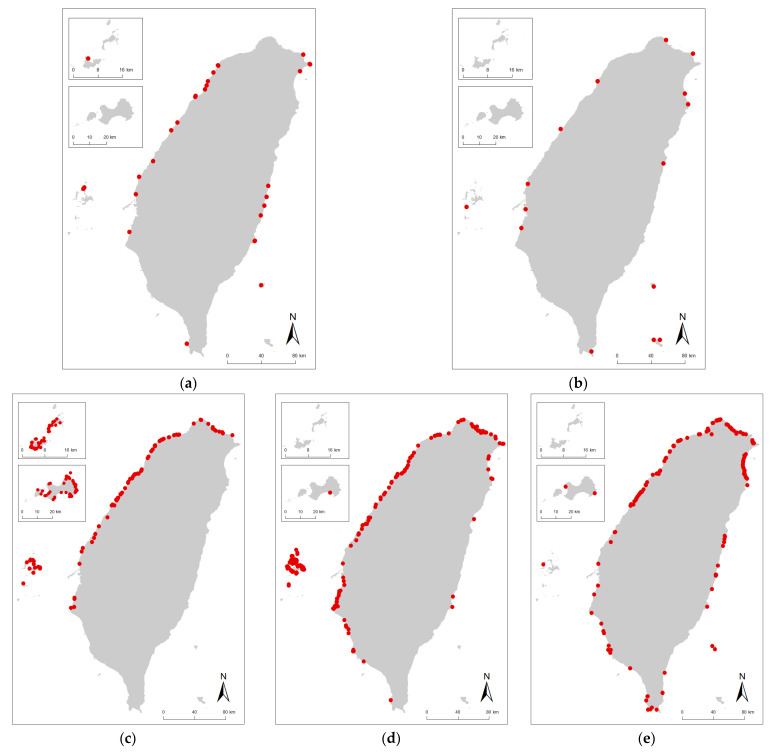
Stranding events (red dots) of various families or species in Taiwan from 1994 to 2018, including (**a**) Balaenopteridae, (**b**) sperm whales, (**c**) finless porpoises, (**d**) bottlenose dolphins, and (**e**) *Kogia* spp.

The distribution patterns of several important species were analyzed and are depicted in Figure 6. Finless porpoises were predominantly stranded in northwestern Taiwan and notably in the isles within the Taiwan Strait. Bottlenose dolphins were primarily found stranded in northern, western, and southern Taiwan, including a specific instance in the Penghu archipelago. *Kogia* spp., known for their deep-diving behaviour, were mainly stranded in northern Taiwan, although occurrences were also noted in the shallow waters of western Taiwan. Sperm whales (*Physeter macrocephalus*), another deep-diving species, were stranded not only in the deep-water areas of eastern Taiwan, but also in the shallow waters of the western region. Baleen whales exhibited similar stranding patterns.

## 4. Live and Mass Stranding

**Figure 7:**
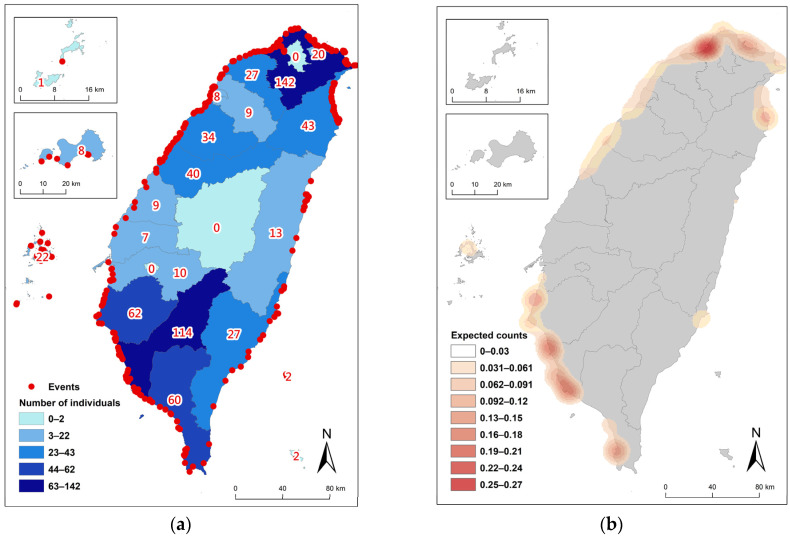
(**a**) The distribution of live stranding events at various counties of Taiwan from 1994 to 2018, totaling 390 events (red dots) and 660 animals (categorized colours and numbers). (**b**) Kernel estimation of live stranding individuals from 1994 to 2018, showing expected counts of stranded individuals per square kilometer.

**Figure 8:**
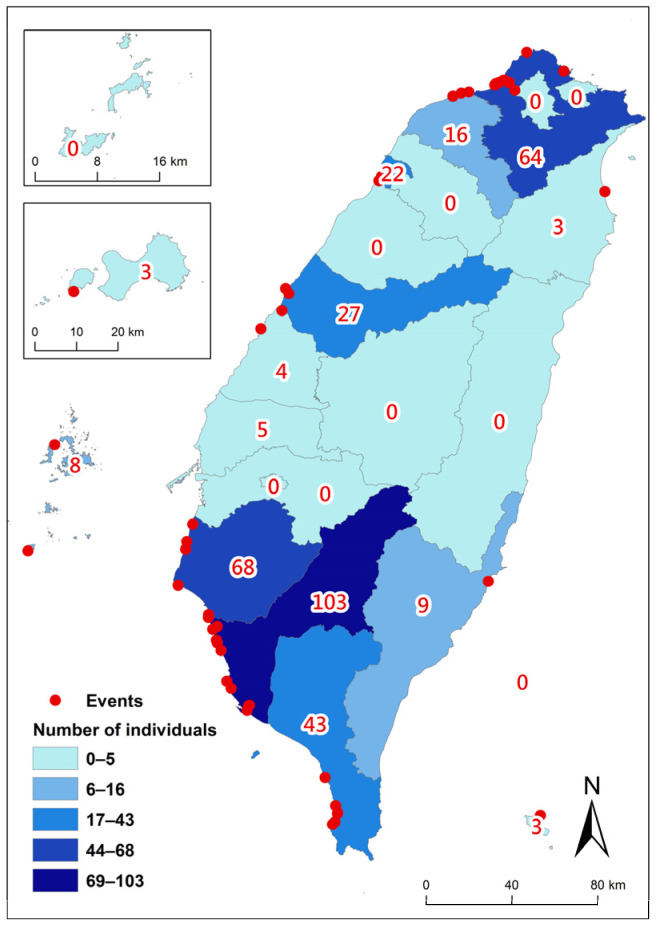
Distribution of mass stranding events in various counties of Taiwan, totaling 56 mass stranding events (red dots) and 378 stranded individuals (categorized colours and numbers) from 1994 to 2018.

The live stranding incidents comprised 390 events and 660 animals, representing 29.5% of the total stranding events and 38.9% of the stranded animals. The distribution of live strandings was not random, with a notably higher proportion in the northern and southwestern areas of Taiwan (Figure 7). Live strandings included single strandings and mass strandings. From 1994 to 2003, both the number of events and individuals show a relatively stable and parallel increase (Figure 2). After 2003, a noticeable discrepancy emerged: the number of individual animals stranded increases significantly compared to the number of events. Peaks in individual strandings are observed in years such as 2005, 2007, 2010, 2011, and 2018, while event counts remain relatively lower in comparison, indicating more mass stranding incidents. There were 56 mass stranding events that occurred, predominantly involving live individuals and spanning at least 11 species. These mass strandings occurred more frequently during the NEM season, accounting for 75% of incidents, and were predominantly located on the northern and southwestern areas of Taiwan (Figure 8), with 89% occurring there if excluding events on the east coast and remote islands. There were twenty-four mass strandings of pygmy killer whales representing 42.9% (24/56) of all mass stranding events, making them the leading species in mass strandings. Most of the events (22/24) occurred from February to April. Moreover, these 24 events accounted for 40.7% of all stranding events of pygmy killer whales.

Efforts to rescue live stranded cetaceans in Taiwan began in 1997. Since 2000, 52 rescue attempts have been made, resulting in the successful rehabilitation and release of 15 individuals. In June 2000, a male Risso’s dolphin (*G. griseus*) was stranded along the west-central coast of Taiwan. After a 64-day rehabilitation program by TCSN, he was released in September, marking the first successful release of a dolphin in the western Pacific after rescue and rehabilitation. He was equipped with a freeze brand and a yellow plastic tag on his dorsal fin for monitoring. Two weeks post-release, he was spotted near Amami Island, Okinawa, approximately 800 km northeast of Taiwan. In 2010, a female subadult pygmy sperm whale (*K. breviceps*) became Asia’s first satellite-tracked rehabilitated cetacean, released into the southwestern waters of Taiwan after a 75-day rehabilitation period; the tracking lasted one month. After this case, two other individuals—one bottlenose dolphin (*T. truncatus*) and one pygmy killer whale (*F. attenuata*)—were also monitored using satellite tracking.

## 5. Discussion

The stranding events in Taiwan, involving at least 27 different cetacean species, underscore the country’s substantial marine biodiversity and its important location along crucial routes in the Western Pacific. Despite its relatively shorter coastline compared to some other studied regions such as Hawaii, Spain, North Carolina, and the Philippines, Taiwan’s cetacean diversity is remarkable. This diversity in the cetacean stranding record exceeds that reported for northwest Spain, Hawaii, San Diego in California, southeastern Canada, and Cape Cod—southeastern Massachusetts (reviewed in [14]). It is comparable to the species diversity observed in North Carolina [14], the Pacific Northwest in the United States [4], various regions in Australia (reviewed in [15]), and the Philippines [16], while being somewhat lower than the diversity documented in Chile [17]. These findings highlight Taiwan’s rich marine biodiversity, despite the country’s comparatively shorter coastline. This ecological complexity is mirrored in the diversity of the stranding records, which is crucial not only for understanding the local biodiversity but also for monitoring the health of wider marine ecosystems [18]. Furthermore, the varied species documented in strandings help highlight the importance of Taiwan’s waters as part of larger biogeographical pathways that are essential for the conservation of marine biodiversity on a global scale [19]. Taiwan’s geographical features, including its location at the convergence of several ocean currents (Figure 1), contribute to complex marine ecosystems which host a wide array of cetacean species ranging from finless porpoises to sperm whales, but may also pose navigational hazards that could contribute to strandings. The stranding patterns suggest that the Taiwan Strait might act as a natural trap, especially for deep-diving species like *Kogia* spp. and sperm whales, which are typically found stranded in relatively shallow waters. The geographic and bathymetric characteristics of the strait could be disorienting or restrictive to these species, suggesting a need for further research into the navigational challenges and environmental barriers posed by this region.

The variety of stranded species could highlight the interactions between cetaceans and multiple environmental, as well as anthropogenic, factors that might influence their survival and behaviours. Stranding events could offer insights into the health and trends of cetacean populations that are otherwise difficult to study in their natural habitats, thus contributing valuable information to the global body of marine mammal science [6,20]. The high incidence of strandings across a diverse set of species reflects the dynamic and sometimes perilous interface between marine organisms and their environment, accentuated by human impacts such as fishing, shipping, and pollution [8]. As such, Taiwan’s stranding data are invaluable not only for regional conservation efforts but also for broader applications in marine ecological research and policy-making.

The relatively high ratio of live strandings in cetaceans observed in Taiwan, where 29.5% of stranding events or 38.9% of stranded animals were live ones, is significantly higher compared to other regions such as Thailand, where only about 9% of stranded individuals were alive [21], and North Carolina, with 6% live strandings [14]. In South Australia, 25.4% of recorded stranding events from 1990 to 2008 involved live animals [22]. The exceptionally high proportion of live strandings in the Philippines, at 60% [23], contrasts with Taiwan’s, and draws attention to regional differences in stranding dynamics and rescue capabilities. Taiwan’s high incidence of live strandings not only indicates the effectiveness of the established cetacean stranding response system but also provides a unique opportunity to advance species-specific veterinary medicine and contributes valuable data on the physiological and behavioural responses of different cetacean species. For example, understanding the nuances in the treatment of a pygmy sperm whale versus a bottlenose dolphin can lead to more effective and species-specific medical care in the future [8,18]. Furthermore, the insights gained from rescuing stranded dolphins and other cetaceans can inform conservation strategies for other endangered species, such as Chinese white dolphins (*Sousa chinensis*) and finless porpoises. Moreover, the establishment of protocols and facilities for cetacean rehabilitation in Taiwan reflects a shift towards more humane and scientifically informed approaches to dealing with cetacean strandings. Effective rescue and rehabilitation efforts, as demonstrated in the first successful recovery and release of a male Risso’s dolphin after a 64-day rehabilitation program, highlight the potential for cetaceans to survive stranding events with human assistance. This dolphin’s case, when it was spotted near Amami Island post-release, marks the success of rehabilitation efforts and the utility of monitoring post-release. These efforts are critical, as they provide valuable data on the survivability and movement patterns of cetaceans post-stranding, contributing to broader conservation science.

The data show a notable increase in annual stranding events, rising from an average of 16 per year in the first three years to 44–58 per year from 1997 to 2015, and surpassing 90 events per year in the last three years [8,18]. The steep increasing trend of cetacean strandings in Taiwan could be particularly intriguing and alarming. It is possible that the apparent increase in strandings is partly due to enhanced monitoring and reporting mechanisms as a result of the established nationwide stranding network mentioned earlier. Over the years, Taiwan has developed a more systematic approach to recording and responding to stranding events. Improved detection and reporting capabilities, coupled with heightened public awareness and institutional support, have likely contributed to the observed rise in stranding incidents [24]. However, the surge in the last three years of the study period suggests that actual incidence rates may be genuinely rising, and some other causing factors could be involved to it. Increased human activities, such as commercial fishing, shipping traffic, and coastal development, are significant factors. Interactions with fishing gear, including bycatch and entanglement, are known to cause considerable cetacean mortality [21,25]. Additionally, the growing intensity of maritime traffic raises the risk of ship strikes, which have been documented as a critical threat to cetaceans [26,27]. These human-induced activities disrupt the natural habitats of cetaceans, leading to higher stranding rates. Marine pollution, including chemical contaminants and plastic debris, poses significant health risks to cetaceans [28,29]. Ingesting plastics and exposure to toxins can lead to physical harm and behavioural changes, increasing the likelihood of strandings [30,31,32]. Anthropogenic sound, including maritime traffic, pile driving, and naval sonar activities, has been recognized as a significant stressor for cetaceans [33,34]. These noises can disrupt communication, navigation, and foraging, increasing the risk of disorientation and stranding. In addition, environmental changes driven by climate change may also be disrupting cetacean habitats and prey availability, potentially increasing the likelihood of strandings. Rising sea temperatures and ocean acidification can alter marine ecosystems, making it difficult for cetaceans to find adequate food sources and safe habitats. For instance, unusual weather patterns and the occurrence of harmful algal blooms (HABs) have been linked to mass mortality events in marine mammals [35]. These environmental stressors can weaken cetaceans, making them more vulnerable to stranding. Taiwan’s coastal waters are not immune to these global issues, and the rising trend in strandings could reflect the cumulative impact of pollution on cetacean health. These factors collectively highlight the urgent need for enhanced monitoring strategies that address both direct and indirect threats to cetacean populations. Assessing anthropogenic activities involves bycatch observation programs, vessel tracking systems, and evaluating modified fishing gear to reduce entanglement risks [25]. Environmental monitoring should include oceanographic stations for tracking changes in water salinity, temperature, and pH, as well as surveillance for prey abundance [35]. Health and biological monitoring through necropsies and biomonitoring programs can detect diseases and toxins affecting cetaceans [34]. Building centralized databases is crucial, and GIS analyses help integrate and visualize data, highlighting stranding hotspots [30,31]. Collaboration among agencies, research institutions, and NGOs is vital for co-ordinating efforts and sharing resources. Training and educational programs can enhance the local capacity for monitoring and conservation efforts. These monitoring strategies provide critical data to inform effective regulations and conservation measures, addressing both direct and indirect threats to cetacean populations.

Furthermore, the proportion of mass strandings in Taiwan is relatively high, accounting for 4.2% of all stranding events. This proportion is higher compared to several studies conducted in other regions. For instance, in Victoria, Australia, only 1.7% of the 424 stranding events were mass strandings [15]. The Philippines reported 48 mass stranding events out of 1368 events, constituting 3.5% of the total from 2005 to 2022 [16]. In Chile, comparably, 17 mass stranding events were recorded among 436 total stranding events, amounting to 3.9% [17]. This higher incidence of mass strandings in Taiwan suggests the presence of unique environmental conditions or human activities that may contribute to these occurrences. Intriguingly, 24 mass stranding cases (42.9%) involved pygmy killer whales. These events predominantly occurred during the NEM season, specifically from February to April, along the southwestern coast of Taiwan. This highlights a clear spatiotemporal pattern in their occurrence. The high frequency of pygmy killer whale mass strandings could be attributed to several factors, including their strong social structure and behaviour, since pygmy killer whales are known for their strong social bonds, which might contribute to mass strandings as individuals follow distressed group members ashore [21]. Environmental factors, such as changes in sea temperature, prey availability, and ocean currents during the NEM season, may also play a role in these strandings [22]. Additionally, human-induced factors, such as potential interactions with specific fishing operations [23] and naval sonar exercises [34], cannot be overlooked, as they may explain the clear spatiotemporal stranding pattern. [23] Given their regular visitation patterns to the southwest coast, it is crucial that we monitor these areas closely and implement measures to mitigate stranding risks.

Finless porpoises were the most frequently stranded cetacean species of the study period in Taiwan, indicating either their high population in the area or their increased susceptibility to stranding. Finless porpoises (genus *Neophocaena*) inhabit shallow waters and become stranded on Taiwan’s west coast and isles in the Taiwan Strait. Two species are found in these waters: the Indo-Pacific finless porpoise (*N. phocaenoides*) and the narrow-ridged finless porpoise (*N. asiaeorientalis*). They appear very similar, with the main difference being the width of the granulated area on their dorsal ridge. Many stranded porpoises are highly decomposed, making species identification difficult for first responders, leading us to combine the stranding numbers of both species. The consistent presence of this genus in the stranding records highlights their vulnerability to various environmental and anthropogenic factors. For instance, finless porpoises often inhabit coastal and shallow waters, making them more prone to human-related threats such as fishing operations, pollution, and habitat degradation [36]. Furthermore, their sensitivity to noise pollution and water quality changes can contribute to higher stranding rates [37]. Given their frequent stranding, targeted research is essential in order to identify specific causes and implement conservation measures to mitigate these risks. Focused monitoring and protection efforts are crucial in order to ensure the survival and well-being of finless porpoises in Taiwanese waters.

The rich dataset from Taiwan serves as a valuable resource for refining global cetacean conservation strategies. The detailed analysis of temporal and spatial stranding patterns, coupled with successful rescue and rehabilitation cases, provides critical insights that can enhance cetacean conservation efforts worldwide. A previous study by [38] examined changing trends in cetacean strandings in the East China Sea, including Taiwan, from 1990 to 2021, by compiling records from news articles, research papers, and other publications. However, only 283 cetacean stranding records of Taiwan were obtained in that study period, highlighting the limitations of ad hoc data collection. In contrast, the current study recorded 1320 stranding events from 1994 to 2018, demonstrating the importance of a well-maintained and systematic stranding database. The significant discrepancy between these numbers emphasizes the necessity of comprehensive and consistent data collection for making accurate and constructive interpretations and suggestions. Maintaining detailed stranding databases allows for the identification of patterns and trends that are crucial for understanding the causes and implications of strandings. These databases enable researchers and conservationists to develop targeted strategies to mitigate stranding risks and enhance the effectiveness of conservation efforts [8,38].

## 6. Conclusions

In conclusion, Taiwan’s cetacean stranding records are characterized by remarkable species diversity and a high incidence of live and mass strandings. These significant findings are credited to a well-established stranding response program and database. Taiwan’s proactive approach to cetacean strandings, supported by robust data collection and a co-ordinated response network, serves as a model for other regions. The stranding response program in Taiwan has been instrumental in advancing conservation practices and veterinary medicine. The cetacean stranding records from Taiwan are invaluable for a deeper understanding of the distribution and habitat use, monitoring population trends and marine ecosystem health, identifying the impacts of both human activities and natural environmental changes, informing management and policy decisions, and, ultimately, improving conservation outcomes on cetaceans in the Western Pacific. The ongoing commitment to research and the implementation of strategic conservation practices are essential for the preservation of these species within marine ecosystems.

## Figures and Tables

**Table 1:** Cetacean species list from 1320 stranding events, including 1698 animals, reported from 1994 to 2018 in Taiwan. * Narrow-edged finless porpoise (*Neophocaena asiaeorientalis*) and wide-edged finless porpoise (*Neophocaena phocaenoides*) were not documented as two species in TCSN database.

Scientific Name	Common Name	No. Stranding Events	No. Stranded Animals
Balaenopteridae			
*Balaenoptera acutorostrata*	Minke whale	6	6
*Balaenoptera edeni*	Bryde’s whale	2	2
*Balaenoptera omurai*	Omura’s whale	9	9
*Megaptera novaengliae*	Humpback whale	2	2
Unidentified Balaenopteridae spp.		10	10
Physeteridae			
*Physeter macrocephalus*	Sperm whale	15	15
Kogiidae			
*Kogia breviceps*	Pygmy sperm whale	55	60
*Kogia sima*	Dwarf sperm whale	106	121
Unidentified *Kogia* spp.		21	25
Ziphiidae			
*Mesoplodon densirostris*	Blainville’s beaked whale	25	25
*Mesoplodon ginkgodens*	Ginkgo-tooth beaked whale	14	19
*Indopacetus pacificus*	Longman’s beaked whale	2	3
*Ziphius cavirostris*	Cuvier’s beaked whale	18	18
Unidentified *Mesoplodon* spp.		7	7
Unidentified Ziphiidae spp.		7	8
Delphinidae			
*Delphinus delphis*	Common dolphin	17	17
*Feresa attenuata*	Pygmy killer whale	59	236
*Globicephala macrorhynchus*	Short-finned pilot whale	28	36
*Grampus griseus*	Risso’s dolphin	86	92
*Lagenodelphis hosei*	Fraser’s dolphin	73	75
*Peponocephala electra*	Melon-headed whale	11	19
*Pseudorca crassidens*	False killer whale	26	26
*Sousa chinensis*	Chinese white dolphin	18	20
*Stenella attenuata*	Pantropical spotted dolphin	73	139
*Stenella coeruleoalba*	Striped dolphin	11	17
*Stenella longirostris*	Spinner dolphin	19	19
*Steno bredanensis*	Rough-toothed dolphin	50	73
*Tursiops aduncus*	Indo-Pacific bottlenose dolphin	28	29
*Tursiops truncatus*	Common bottlenose dolphin	152	154
Unidentified *Tursiops* sp.		12	12
Unidentified Delphinidae sp.		59	81
Phocoenidae			
*Neophocaena* spp. *	Finless porpoise	228	235
Unidentified sp.		71	88

## Data Availability

The original contributions presented in the study are included in the article, further inquiries can be directed to the corresponding author.
